# In Vitro Ruminal Fermentation and Methane Output of Monospecific, Binary, and Multispecies Pastures Under Two Defoliation Frequencies

**DOI:** 10.3390/ani16152396

**Published:** 2026-08-03

**Authors:** Isidora P. Ruiz-Tagle-Renner, Juan P. Keim, Oscar A. Balocchi, Iván Calvache

**Affiliations:** 1 Graduate School, Faculty of Agricultural and Food Sciences, Universidad Austral de Chile, Independencia 641, Valdivia 5110566, Chile; isidora.ruiz-tagle@alumnos.uach.cl; 2 Animal Production Institute, Faculty of Agricultural Sciences, Universidad Austral de Chile, Independencia 641, Valdivia 5110566, Chile; obalocch@uach.cl; 3 Institute of Animal Science, School of Veterinary Sciences, Universidad Austral de Chile, Independencia 641, Valdivia 5110566, Chile; ivan.calvache@uach.cl

**Keywords:** multispecies pastures, defoliation frequency, growing degree days, in vitro fermentation, methane, volatile fatty acids, ruminal ammonia

## Abstract

Pastures are the dietary basis of grazing systems in regions such as southern Chile, and their botanical composition and management can influence ruminal fermentation and associated by-products. The objective of this study was to evaluate the effects of pasture type and defoliation frequency (DF) on in vitro ruminal fermentation kinetics and methane and ammonia production. Four pasture types were evaluated: *Bromus valdivianus* Phil. monoculture (Bv), *Lolium perenne* monoculture (Lp), a binary mixture of both species (LpBv), and a multispecies pasture (Msp). All pastures were managed under two defoliation frequencies (150 and 300 growing degree days [GDDs]) and sampled across four seasons (summer, autumn, winter, and spring). Samples were dried at 60 °C, ground, and incubated for 48 h at 39 °C using the ANKOM RF automated gas production system, with ruminal inoculum collected from rumen-cannulated cows. Total gas production, methane production, and concentrations of volatile fatty acids (VFAs) and ammonia (NH_3_) were evaluated. The CH_4_ proportion was affected by the DF × season interaction, and CH_4_ intensity was affected by the pasture type × DF interaction. Total gas production was greater for Lp than for Bv and Msp and was greater at 300 than at 150 GDD. Total VFAs showed a pasture type × DF × season interaction. Across pasture types, NH_3_ concentration was lower at 300 than at 150 GDD. Overall, no treatment consistently reduced methane output. Extending the defoliation interval may reduce ruminal NH_3_, but its environmental benefit should be evaluated together with seasonal methane responses and potential trade-offs in forage nutritive value and animal performance.

## Simple Summary

Livestock production systems face the dual challenge of increasing output while reducing environmental impact, particularly greenhouse gas emissions and nitrogen losses. This study evaluated how pasture type and defoliation frequency affected in vitro ruminal fermentation, methane (CH_4_), and ammonia (NH_3_) production. Defoliation frequency affected CH_4_ production per gram of incubated dry matter and NH_3_ concentration, whereas pasture type primarily affected total gas production. Across pasture types, the longer defoliation interval of 300 growing degree days (GDDs) reduced NH_3_ concentration. Methane responses were less consistent: no pasture treatment consistently reduced CH_4_ output, and significant responses depended on pasture type and season. Thus, extending the defoliation interval may reduce potential ruminal nitrogen losses, but this benefit should be considered together with changes in forage nutritive value and the absence of consistent methane mitigation.

## 1. Introduction

Animal production must meet the growing demand for livestock products associated with global population growth [1] while adopting sustainable practices that reduce its environmental impact [2]. Livestock farming contributes approximately 14.5% of anthropogenic greenhouse gas (GHG) emissions, including carbon dioxide (CO_2_), methane (CH_4_), and nitrous oxide (N_2_O) [3]. It is also a major source of NH_3_, an indirect precursor of N_2_O, which has a global warming potential 265 times greater than that of CO_2_ [4]. Methane represents approximately 44% of livestock GHG emissions and has a global warming potential 28 times greater than that of CO_2_. In ruminants, it is mainly produced during enteric fermentation as rumen microorganisms degrade carbohydrates into volatile fatty acids (VFAs) [4]. Dietary nitrogen is also metabolized to NH_3_, which can be used for microbial protein synthesis. However, when NH_3_ availability exceeds the supply of fermentable energy, it accumulates in the rumen, is absorbed and converted to urea in the liver, and is either recycled to the rumen or excreted mainly in urine, thereby contributing indirectly to N_2_O emissions [5].

Because CH_4_ production and N excretion are products of ruminal fermentation, both may be mitigated through dietary modulation of rumen metabolism. Strategies such as feed additives, algae, vegetable oils, and concentrates have been investigated, but their implementation is difficult in grazing systems such as those predominant in southern Chile [6], where pasture is the main dietary component [7].

A promising mitigation strategy is therefore to modify pasture botanical composition by incorporating species rich in plant secondary metabolites or soluble sugars. For example, high-sugar *Lolium perenne* cultivars managed with lower grazing frequency and reduced nitrogen fertilization have been shown to decrease ruminal ammonia concentrations and CH_4_ production [6]. However, pastures dominated by *L*. *perenne* combined with *T*. *repens* or *B*. *valdivianus* have not consistently reduced CH_4_ production [8], indicating that species selection is critical for optimizing rumen fermentation and mitigating environmental impacts. In this context, multispecies swards (MSSs) combining grasses, legumes, and broadleaf forbs represent a promising alternative [9].

Species diversity can stabilize annual dry matter (DM) production and improve pasture nutritional value [9]. Legumes such as *Trifolium pratense* and *T*. *repens* fix atmospheric nitrogen, reducing fertilizer requirements [10], whereas forbs such as *Plantago lanceolata* and *Cichorium intybus* contain plant secondary metabolites (PSMs), including tannins and saponins, that may reduce CH_4_ production or improve nitrogen use efficiency [11]. Grasses such as *L*. *perenne* and *B*. *valdivianus* contribute high forage yield, quality, and adaptability [12,13].

Multispecies pastures may alter rumen metabolism through differences in crude protein, fiber concentration, and organic matter digestibility [14], as well as through bioactive compounds that affect microbial activity, nitrogen partitioning, and enteric CH_4_ production [15,16]. However, in vitro studies have produced inconsistent results. Mixtures containing plantain and red clover reduced CH_4_ production and NH_3_ concentration by 14.7% and 28.8%, respectively, relative to a fertilized perennial ryegrass–red clover control [17]. Conversely, the fermentation response of a six-species mixture generally did not differ from the average response of its component monocultures, suggesting that mixture effects may be predicted from botanical composition [18]. Similarly, diverse pastures evaluated in southern Chile did not affect CH_4_ production, although pasture type interacted with regrowth period to influence ammonia and microbial nitrogen [8].

Seasonal responses have also been reported. Compared with ryegrass–white clover pasture, plantain-containing pasture reduced NH_3_ production in spring and autumn, whereas CH_4_ reductions occurred only in summer [17]. Moreover, although 19 of 35 herbaceous species reduced in vitro ammonia concentration, 18 also decreased organic matter digestibility, indicating potential trade-offs in fermentation efficiency [19]. Overall, the effects of diverse pastures on CH_4_ and NH_3_ production depend on botanical composition, the presence and proportion of species containing bioactive metabolites, and harvest season [17,20].

Defoliation frequency is therefore a major determinant of MSS composition and function [21]. Frequent defoliation may improve nutritive value but reduce DM yield and the persistence of sensitive species, particularly forbs, whereas longer intervals increase biomass but reduce forage quality through greater lignification [22,23]. Adaptive defoliation strategies that account for species-specific growth dynamics are therefore required to maintain functional diversity and pasture performance. Therefore, the combination of appropriate species and management could provide an optimal nutrient balance, thereby improving rumen metabolism and fermentation kinetics [24].

Thus, the objective of this study was to evaluate the effects of monospecific *L. perenne* and *B. valdivianus* pastures, a binary *L. perenne*–*B. valdivianus* mixture (LpBv), and a multispecies pasture (Msp) managed under two defoliation intervals (150 and 300 GDD) across the four seasons of the year on rumen metabolism and in vitro fermentation kinetics. This approach aimed to determine how pasture type and defoliation management influence fermentation efficiency and environmentally relevant ruminal outputs. We hypothesized that the multispecies pasture managed at the longer defoliation interval (300 GDD) would reduce in vitro CH_4_ production and NH_3_ concentration because broadleaf-derived plant secondary metabolites may inhibit or modulate methanogenesis and proteolysis, while the higher water-soluble-carbohydrate-to-crude-protein ratio expected at longer regrowth intervals may favor microbial capture of ruminal NH_3_.

## 2. Materials and Methods

This study was conducted as part of FONDECYT Regular Project (1220448), “*Bromus valdivianus* Phil: contribution to biodiversity and sustainability of pasture-based livestock systems.” The study was conducted using an in vitro fermentation system at the Animal Nutrition Laboratory of the Universidad Austral de Chile, and experimental plots were established at the Austral Agricultural Research Station (EEAA) located at 39°46′28″ S, 73°14′11″ W. The procedures performed on animals in this trial were approved by the Institutional Committee for Animal Care and Use of the Universidad Austral de Chile (468/2022).

### 2.1. Establishment of the Pastures

Experimental pastures were established in August 2022 at the EEAA in 20 m^2^ plots (4 m × 5 m), arranged in a completely randomized block design with three blocks. The fertilizer application rate was 600 kg ha^−1^ of 05-28-16 (N-P-K) blend, 1000 kg ha^−1^ of CaCO_3_, and 350 kg ha^−1^ of MgO. Seeding rates were determined according to species-specific recommendations. The monospecific *L*. *perenne* pasture (Lp) was seeded at a rate of 30 kg ha^−1^, while the *B*. *valdivianus* pasture (Bv) was seeded at 45 kg ha^−1^. For the binary pasture (LpBv), rates of 15 and 30 kg ha^−1^ were used, respectively. The multispecies pasture (Msp) was established with 5.1 kg ha^−1^ of *L*. *perenne*, 10.2 kg ha^−1^ of *B*. *valdivianus*, 1.7 kg ha^−1^ of *T*. *repens*, 3.4 kg ha^−1^ of *T*. *pratense*, 1.7 kg ha^−1^ of *P*. *lanceolata*, and 1.7 kg ha^−1^ of *C*. *intybus*, ensuring uniform species distribution within each plot.

### 2.2. Pasture Sampling

The four pasture types (Lp, Bv, LpBv and Msp) were harvested under two defoliation frequencies (DFs) defined on the basis of thermal time, expressed as growing degree days (GDDs), 150 and 300 GDD. Pastures were assessed across the four seasons of the year.

**Figure 1:**
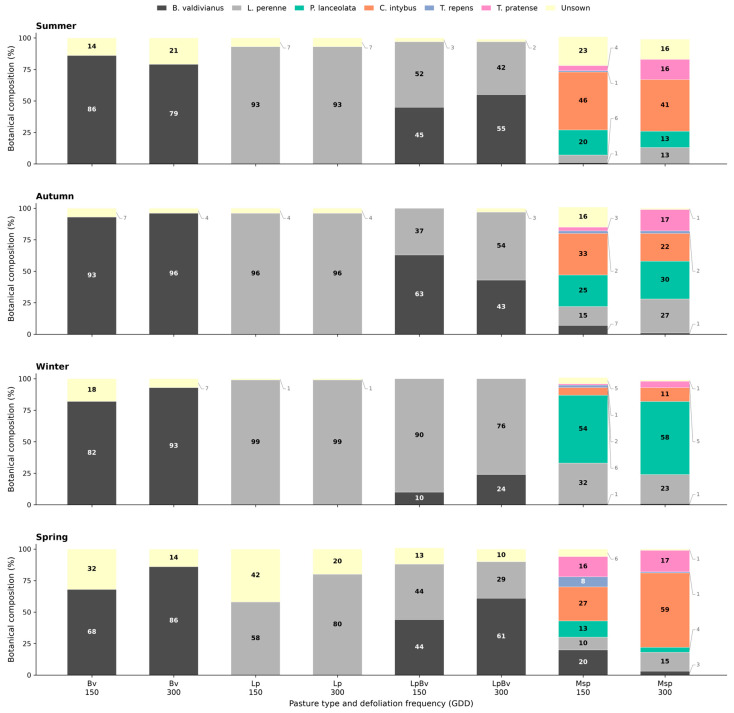
Botanical composition of the pastures evaluated under two defoliation frequencies across the four seasons of the year. Bv: *B. valdivianus* monoculture; Lp: *L. perenne* monoculture; LpBv: binary pasture of *L. perenne* and *B. valdivianus*; Msp: multispecies pasture. The values 150 and 300 represent defoliation frequencies at 150 and 300 GDD.

Pasture samples for incubation were collected following the established sampling design (150 and 300 GDD) during each season of 2023. Samples were collected on January 18 (summer), April 22 (autumn), July 4 (winter), and November 6 (spring). Prior to each harvest, the botanical composition of each plot was determined, as presented in Figure 1. For this purpose, 150 g forage subsamples were collected at ten random positions using a 0.04 m^2^ quadrat within each plot. In the laboratory, samples were manually separated by species to determine the relative proportion of sown and unsown species as a percentage of the total collected biomass.

Before collecting the pasture samples for incubation, the first 50 cm along the perimeter of each plot were excluded to avoid border effects; the remaining area (12 m^2^) was then mowed with a rotary mower (LC 140P, Husqvarna, Huskvarna, Jönköping, Sweden) to a residual height of 5 cm, to preserve plant reserve structures and simulate grazing conditions. After collection, forage samples were oven-dried at 60 °C for 72 h to constant weight, ground to pass through a 1 mm screen, and stored in sealed containers at room temperature until incubation. The concentrations of crude protein (CP), metabolizable energy (ME), neutral detergent fiber (NDF), acid detergent fiber (ADF), soluble protein (SP), water-soluble carbohydrates (WSCs), and digestibility value (DV) were subsequently determined by near-infrared spectroscopy (NIRS). A FOSS-NIRSystems MODEL 6500 (FOSS NIRSystem Inc., Silver Spring, MD, USA) was used with prediction equations developed from wet chemistry results by the Animal Nutrition Laboratory of the Universidad Austral de Chile. The standard errors of cross-validation were 0.78, 1.92, 1.19, 0.30, and 6.99 for CP, NDF, ADF, ME, and WSC, respectively, while the R2 values were 0.98, 0.93, 0.93, 0.84, and 0.96, respectively.

### 2.3. In Vitro Incubation

The in vitro incubation method described by Theodorou et al. [25] was used, with modifications for the ANKOM RF automated gas production system (ANKOM Technology, Macedon, NY, USA), which enables automatic recording and venting of fermentation gas.

One day before incubation, triplicate 1500 mg samples of each pasture were weighed with a precision balance PRACTUM101-1S (Sartorius Lab Instruments GmbH & Co. KG, Göttingen, Germany) and added into 250 mL incubation vessels. In addition, triplicate 1500 mg samples of a standard concentrate and one blank vessel were incubated. A total of 120 mL of Goering–Van Soest incubation buffer was added to each vessel; the buffer had previously been purged with CO_2_ for two hours to ensure anaerobic conditions. Each vessel was then flushed with CO_2_ for 10 s, sealed, and refrigerated at 4 °C overnight. Shortly before inoculation, 6 mL of reducing solution was added to each vessel, followed by a 40 mL CO_2_ flush. Vessels were placed in a water-bath incubator (GFL1083; GFL, Burgwedel, Germany) at 39 °C in a randomized arrangement. Rumen inoculum was collected before morning feeding from two rumen-cannulated, lactating Holstein-Friesian cows maintained at the EEAA (milk yield: 16.2 ± 1.5 L per day; days in milk: 240 ± 35; body weight: 495 ± 8.5 kg). Approximately 1 L of rumen fluid was collected from each cow, strained into pre-warmed thermos flasks, and transported promptly to the Animal Nutrition Laboratory. Rumen fluid from each donor cow was filtered separately twice through a synthetic fabric mesh under CO_2_-saturated conditions at 39 °C. Equal volumes of filtered rumen fluid from the two cows were pooled to obtain a single inoculum for each incubation run, and 30 mL of the pooled inoculum was added to each vessel. The ANKOM RF system was configured with a live communication interval of 60 s and a data-recording interval of 5 min; automatic gas release occurred when module pressure reached 0.8 psi. Twelve incubation runs were performed, with one field block incubated per week and three blocks incubated per season. A standard concentrate with a known fermentation profile was included in triplicate in every run as an internal quality-control substrate and was not included in the statistical analysis of pasture treatments. To assess between-run consistency, vessel-level cumulative gas-production values for the standard concentrate were analyzed using a one-way linear model with incubation run as a fixed effect. No evidence of differences among runs was detected for 48 h cumulative gas production (*p* > 0.10).

### 2.4. Sampling of CH_4_, VFAs, NH_3_, and Residue

After 4, 12, 24, and 48 h of incubation, gas samples were collected from each module to determine CH_4_ concentration. Briefly, 4 mL of headspace gas was extracted from each module and injected into 5.9 mL Exetainer tubes; gas from the three technical replicate vessels corresponding to each field-plot sample was combined in a single vial at each sampling time. Samples were analyzed by gas chromatography (PerkinElmer Clarus 590 GC, Waltham, MA, USA) using an Elite GC GS Molesieve column (PerkinElmer Inc., Waltham, MA, USA), with N_2_ as the carrier gas at 1 mL min^−1^. The manual injection volume was 200 µL. Injector and detector temperatures were set to 200 °C, and the oven temperature was held at 170 °C for 14 min. Methane was quantified using an external calibration curve derived from the linear regression of chromatographic peak areas.

At the end of the incubation, vessels were opened, covered with Parafilm, and stored at −20 °C for 1 h to arrest fermentation. The contents of the three technical replicate vessels corresponding to each field-plot sample were pooled and homogenized in a plastic container. Ten milliliters of supernatant were withdrawn for VFA analysis and an additional 10 mL for NH_3_ analysis. The pooled material was also used for pH and residue-based degradability determinations. Pooling yielded one representative analytical observation per experimental unit and reduced the number of laboratory determinations required; consequently, vessel-level variability could not be estimated for VFAs, NH_3_, pH, or degradability.

After extraction of the supernatant, the wet residue was weighed and its pH measured using a pH meter (Thermo Scientific Orion Star A214, Waltham, MA, USA); the residue was then quantitatively recovered from the plastic containers using distilled water. The residue was oven-dried at 60 °C to constant weight, weighed, and analyzed for DM and organic matter (OM) content to calculate in vitro nutrient degradability using the following equation:

(1) $$D_{x}=100-\left(\frac{Res\;*\;X_{res}}{gInc\;*\;X_{p}}\right)$$

where $D_{x}$ represents the percentage of nutrient degradability, *Res* is the amount of residue (g), $X_{res}$ is the nutrient concentration in the residue (%), gInc is the amount of incubated pasture (g), and $X_{p}$ is the nutrient concentration in the pasture (%).

Supernatants were stored at −20 °C in Falcon tubes containing 0.6 mL of orthophosphoric acid (H_3_PO_4_) as a preservative. Prior to analysis, samples were thawed, transferred to 2 mL microtubes, centrifuged at 14,000× *g* for 10 min (MIKRO 200 centrifuge, Andreas Hettich GmbH & Co. KG, Tuttlingen, Baden-Württemberg, Germany), and filtered through a 0.22 µm membrane filter into 1.5 mL microtubes. For VFA determination, 500 µL of each subsample was diluted in chromatography vials with 800 µL isopropanol (Merck KGaA, Darmstadt, Hesse, Germany) and 200 µL 24% v/v H_3_PO_4_ (Merck KGaA, Darmstadt, Hesse, Germany). Volatile fatty acid (acetate, propionate, butyrate, valerate, isobutyrate and isovalerate) concentrations were determined by gas chromatography with flame ionization detection (FID; PerkinElmer Clarus 590 GC, Waltham, MA, USA), using a 35 m ELITE-FFAP column (PerkinElmer, Waltham, MA, USA) and N_2_ as the carrier gas at 1 mL min^−1^. The oven temperature program was 103 °C for 1 min, followed by a ramp of 6 °C min^−1^ to 200 °C, held for 3 min. Injector and detector temperatures were set to 220 °C and 230 °C, respectively. One µL of each sample was injected with a split ratio of 1:20. Each VFA was quantified using an individual calibration curve, with concentrations calculated from the linear regression of chromatographic peak areas. Ammonia concentration was determined colorimetrically by the phenol–hypochlorite method [26], using a UV-Vis spectrophotometer UV-1800 (Shimadzu Corporation, Kyoto, Kyoto Prefecture, Japan).

### 2.5. Calculations

Cumulative gas production curves were fitted to the multiphasic (three phases) sigmoid model described by Groot et al. [27] to estimate gas production during each fermentation phase of the substrate, using the following equation:

(2) $$G\left(t\right)=\sum_{i=1}^{n}\frac{A_{i}}{1+\frac{B_{i}^{C_{i}}}{t^{C_{i}}}}$$

where G is the cumulative gas production (mL g^−1^ OM) at incubation time *t*; $A_{i}$ is the asymptotic gas production of phase i (mL g^−1^ OM); $B_{i}$ is the time required to reach 50% of asymptotic gas production ($A_{i}$); $C_{i}$ is a constant that determines the shape of the curve; and i represents the number of phases.

To calculate the volume of CH_4_ produced, the method used by Tavendale et al. [28] was employed:

(3) $${CH}_{4}\left(mL\right)\;at\;X=\left[{CH}_{4}\%\left(x\right)-{CH}_{4}\%(x-1)\right]\;*\;HSP/100+{CH}_{4}\%\left(x\right)\;*\;GV(x)/100$$

where X represents time (h); $\left(x-1\right)$ represents the previous incubation time; HSP represents the free volume (mL) of the bottle; and GV is the volume of gas produced (mL).

The CH_4_ production was standardized for incubated and digested *DM* and *OM* using the following formulas:

CH_4_ (mL) per gram of incubated *DM*:

(4) $${CH}_{4}\left(mL\;g^{-1}{DM}_{inc}\right)=\frac{{CH}_{4}(mL)}{g\;{DM}_{inc}}$$

CH_4_ (mL) per gram of digested *DM*:

(5) $${CH}_{4}\left(mL\;g^{-1}{DM}_{dig}\right)=\frac{{CH}_{4}\;g^{-1}{DM}_{inc}}{\left(DMD/100\right)}$$

CH_4_ (mL) per gram of incubated *OM*:

(6) $${CH}_{4}\left(mL\;g^{-1}{OM}_{inc}\right)=\frac{{CH}_{4}(mL)}{g\;{OM}_{inc}}$$

CH_4_ (mL) per gram of digested *OM*:

(7) $${CH}_{4}\left(mL\;g^{-1}{OM}_{dig}\right)=\frac{{CH}_{4}\;g^{-1}{OM}_{inc}}{\left(OMD/100\right)}$$

where DMD represents dry matter digestibility (%) and OMD represents organic matter digestibility (%).

### 2.6. Experimental Design and Statistical Analysis

A randomized complete block design with a 4 × 2 factorial arrangement was used, comprising four pasture types (Lp, Bv, LpBv, and Msp) and two defoliation intervals. Season was treated as a repeated measure. Models were fitted using restricted maximum likelihood (REML), and denominator degrees of freedom were calculated using the Kenward–Roger method. The variance–covariance structure with the lowest corrected Akaike information criterion (AICc) was compound symmetry and was selected for the final repeated-measures model.

The assumptions of normality and homogeneity of variance were assessed using the Shapiro–Wilk and Levene’s tests, respectively, together with visual inspection of quantile–quantile plots and residuals versus fitted values. No substantial violations of model assumptions were detected.

The experimental unit was the individual field plot corresponding to each pasture type × defoliation frequency combination within a field block. The three vessels incubated for each field-plot sample were technical replicates rather than independent experimental units. For gas-production kinetics recorded separately in each vessel, the mean of the three technical replicates was used as the observation for the corresponding field plot and season. For CH_4_ determination, gas from the three technical replicate vessels was combined at each sampling time, yielding one observation per field plot, season, and sampling time. For VFAs, NH_3_, pH, and degradability, the fermentation contents and residues from the three vessels were pooled at the end of incubation, yielding one analytical observation per field plot and season.

Statistical analyses were performed using SAS v. 9.4 software (SAS Institute Inc., Cary, NC, USA). Data were analyzed using linear mixed models, with pasture type, defoliation frequency, season, and their interactions included as fixed effects. Field block was included as a random effect, and season was modeled as a repeated measure on the same field plot, defined by the block × pasture type × defoliation frequency combination. Because one field block was incubated per run within each season, incubation run and field block within season were confounded and were therefore not included as separate random effects.

The following statistical model was used:

$$Y_{ijkl}=µ+\beta_{i}+\alpha_{j}+\gamma_{k}+\delta_{l}+{\alpha \gamma }_{jk}+{\alpha \delta }_{jl}+{\gamma \delta }_{kl}+{\alpha \gamma \delta }_{jkl}+{Ɛ}_{ijkl}$$

where $Y_{ijkl}$ is the response variable; µ is the overall mean; $\beta_{i}$ is the block effect; $\alpha_{j}$ is the effect of pasture type; $\gamma_{k}$ is the effect of defoliation frequency; $\delta_{l}$ is the effect of season; ${\alpha \gamma }_{jk}$ is the interaction effect between pasture type and defoliation frequency; ${\alpha \delta }_{jl}$ is the effect of the interaction between pasture type and season; ${\gamma \delta }_{kl}$ is the effect of the interaction between defoliation frequency and season; ${\alpha \gamma \delta }_{jkl}$ is the three-way interaction among the evaluated factors; and ${Ɛ}_{ijkl}$ is the random error. When significant effects were detected (*p* < 0.05), mean comparisons were performed using Tukey’s test for the main effects and Bonferroni-adjusted comparisons for interactions.

## 3. Results

### 3.1. Chemical Composition of Pastures

The chemical composition and nutritional value of the incubated pastures were significantly influenced by the interactions between pasture type and defoliation frequency (P × DF), pasture type and season (P × S), and defoliation frequency and season (DF × S), although no significant three-way interaction was detected (*p* > 0.05). Given that seasonal variation is inherent in this type of study, season was included as a repeated measure. As shown in Table 1, the interaction between pasture type and defoliation frequency (P × DF) significantly affected crude protein (CP; *p* = 0.003), acid detergent fiber (ADF; *p* < 0.001), soluble protein (SP; *p* < 0.001), and water-soluble carbohydrates (WSC; *p* = 0.010).

Crude protein exhibited a pasture type-dependent response to defoliation frequency (P × DF). In the monocultures (Lp and Bv) and the binary pasture (LpBv), CP was significantly higher under more frequent defoliation (150 GDD) than under less frequent defoliation (300 GDD), increasing from 17.5% to 23.0% in Bv, from 15.9% to 20.7% in Lp, and from 16.8% to 21.5% in LpBv. In contrast, Msp showed no difference in CP between intervals. Soluble protein followed the same pattern. NDF was not affected by the P × DF interaction (*p* = 0.107) but was affected by pasture type and defoliation frequency (*p* < 0.001); overall, NDF was greater at the longer interval of 300 GDD. ADF showed a P × DF interaction (*p* < 0.001), increasing from 150 to 300 GDD in LpBv and Bv but not in Lp or Msp. WSC also showed a P × DF interaction (*p* = 0.010), with the highest concentration in Lp at 300 GDD, whereas Msp remained similar across intervals. Metabolizable energy and DV were affected by pasture type but not by P × DF, and Bv had the lowest values. Ash was greater at 150 than at 300 GDD (*p* = 0.023).

### 3.2. In Vitro Fermentation Kinetics

In vitro fermentation kinetics were influenced by pasture type, defoliation frequency, and season across the three fermentation phases. Pasture type × defoliation frequency interactions were detected for k2 (*p* = 0.044) and A3 (*p* = 0.049), and a pasture type × season interaction was detected for k1 (*p* = 0.003; Table 2).

**Figure 2:**
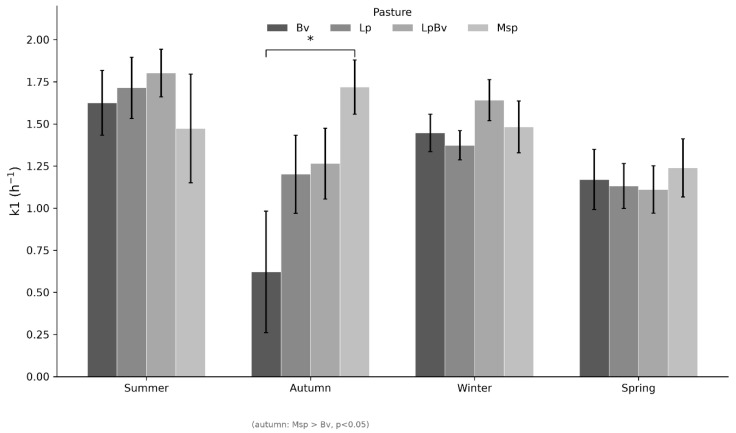
Interaction between pasture type and season on the fermentation rate of soluble carbohydrates (k1). Bv: *Bromus valdivianus*; Lp: *Lolium perenne*; LpBv: binary pasture; and Msp: multispecies pasture; The horizontal bracket and asterisk indicate a significant pairwise difference between the connected pasture types within autumn (*p* < 0.05). Error bars represent standard errors of the least-squares means.

In the first phase, associated with the fermentation of soluble carbohydrates, A1 was significantly affected by pasture type (*p* = 0.016) and season (*p* < 0.001). The Lp pasture recorded the highest value (35.53 mL g^−1^ DM), while Bv showed the lowest (22.42 mL g^−1^ DM), with LpBv and Msp at intermediate values. The fermentation rate (k1) showed effects of pasture type (*p* = 0.027), DF (*p* = 0.034), season (*p* < 0.001), and a P × S interaction (*p* = 0.003). Pastures managed at 150 GDD exhibited a higher fermentation rate (1.45 h^−1^) than those at 300 GDD (1.25 h^−1^), whereas the P × S interaction (Figure 2) revealed generally similar values across pasture types within each season; however, in autumn, differences were detected, with Msp recording the highest k1 value and Bv the lowest. The lag time of the first phase (L1) was influenced by pasture type (*p* = 0.014) and season (*p* < 0.001). The LpBv pasture exhibited the longest lag time (0.71 h), which was significantly greater than that of Bv and Msp (0.45 and 0.44 h, respectively).

**Figure 3:**
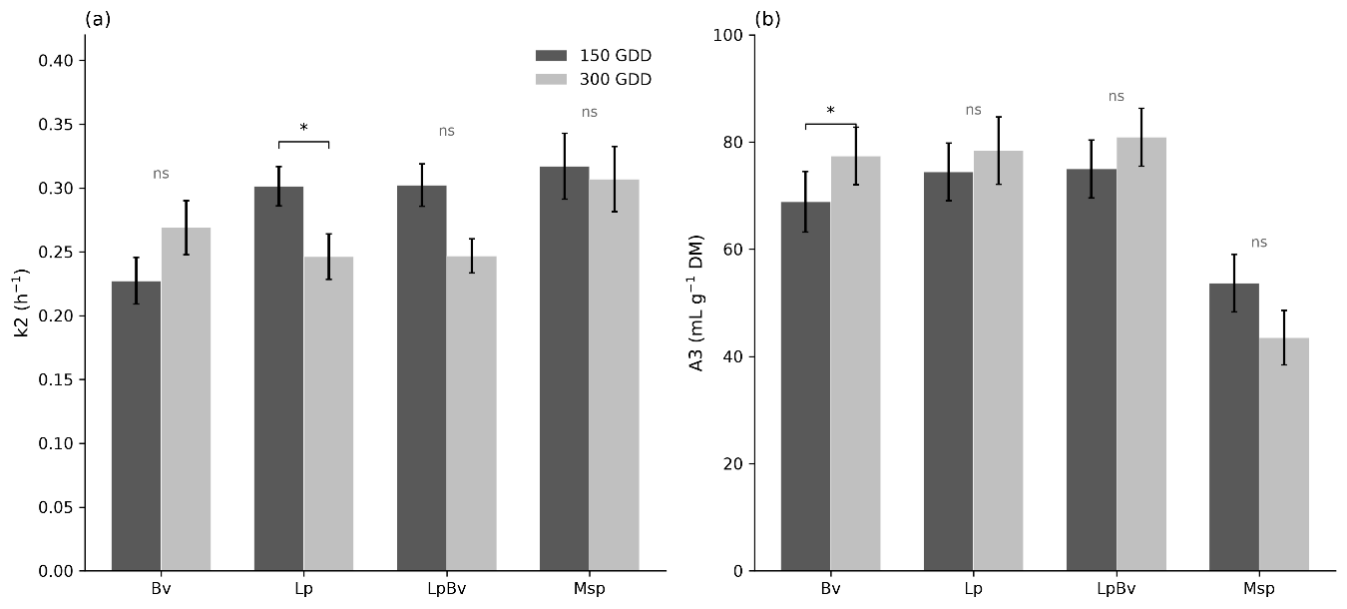
Interactions between pasture type and defoliation frequency on kinetic parameters. (**a**) Fermentation rate of rapidly available fiber (k2). (**b**) Gas production from slowly available fiber (A3). Bv: *Bromus valdivianus*; Lp: *Lolium perenne*; LpBv: binary pasture; Msp: multispecies pasture; GDD: growing degree days. The horizontal bracket and asterisk indicate a significant pairwise difference between the connected pasture types within autumn (*p* < 0.05). ns indicate non significant differences (*p* > 0.05). Error bars represent standard errors of the least-squares means.

In the second phase, representing the fermentation of rapidly degradable fiber, asymptotic gas production (A2) was affected by season only (*p* = 0.041). The fermentation rate (k2) showed a significant P × DF interaction (*p* = 0.044; Figure 3a), indicating that fermentation was faster at 150 GDD in the Lp pasture, while k2 in LpBv, Msp, and Bv was similar across defoliation frequencies, although Bv tended to respond in the opposite direction. The lag time (L2) showed significant effects of pasture type (*p* = 0.025) and season (*p* < 0.001). LpBv had the longest lag time (4.85 h), significantly greater than Msp (3.75 h), while Bv and Lp had intermediate values.

In the third phase, gas production from slowly degradable fiber (A3) showed significant effects of pasture type (*p* = 0.006), season (*p* < 0.001), and a P × DF interaction (*p* = 0.049). The P × DF interaction (Figure 3b) revealed a significant difference between defoliation frequencies for the Bv pasture, whereas no differences were observed between DF levels for the Lp, LpBv, and Msp pastures. The fermentation rate of slowly degradable fiber (k3) was significantly affected by season (*p* < 0.001), with winter recording the highest value (0.12 h^−1^), significantly greater than spring (0.10 h^−1^) and autumn (0.11 h^−1^). Finally, the lag time of the third phase (L3) was significantly influenced by pasture type (*p* = 0.044), with Msp recording the shortest lag time (12.91 h) and Bv the longest (14.51 h).

### 3.3. Ammonia and Volatile Fatty Acids

**Figure 4:**
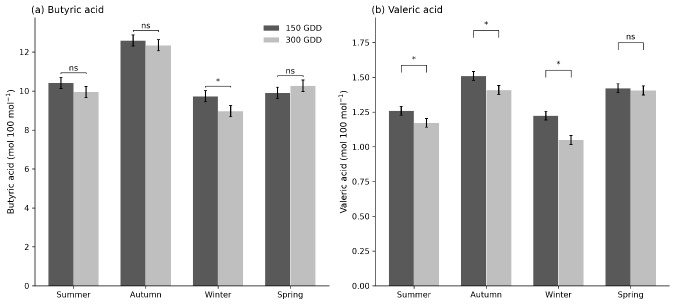
Interaction between defoliation frequency and season on the individual proportions of the volatile fatty acids butyric (**a**) and valeric (**b**). GDD: growing degree days. Within each season, an asterisk indicates a significant difference between 150 and 300 GDD (*p* < 0.05); ns indicates no significant difference. Error bars represent standard errors of the least-squares means.

**Figure 5:**
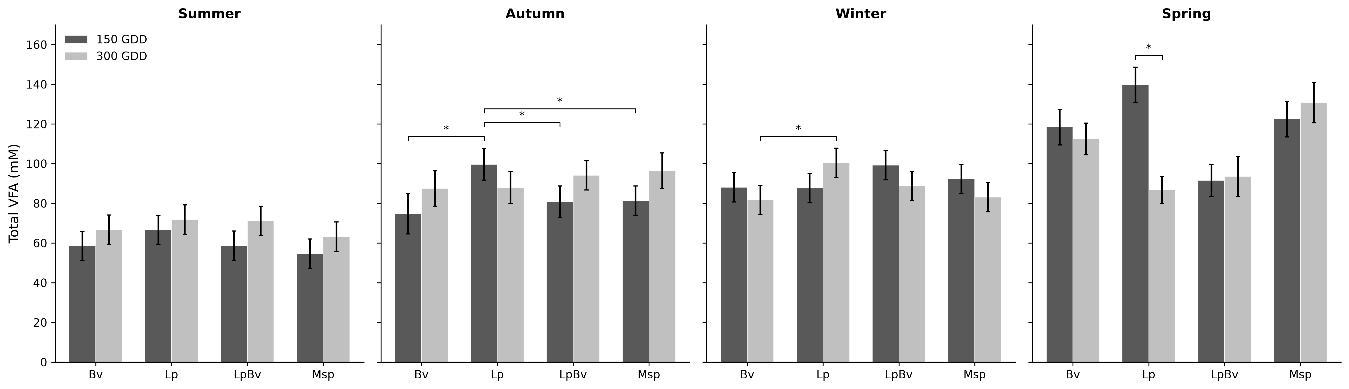
Interaction between pasture type, defoliation frequency, and season on total volatile fatty acid concentration in summer, autumn, winter, and spring. Bv: *Bromus valdivianus*; Lp: *Lolium perenne*; LpBv: Lp and Bv mixture; Msp: multispecies; GDD: growing degree days. Horizontal brackets and asterisks indicate significant pairwise comparisons between the connected least-squares means within the corresponding season and defoliation frequency (*p* < 0.05). Error bars represent standard errors of the least-squares means.

Individual VFA proportions and total VFA concentration were strongly influenced by season (Table 3). Pasture type affected the proportions of acetate, propionate, isobutyrate, and isovalerate, whereas defoliation frequency affected butyrate, valerate, isobutyrate, and isovalerate. Defoliation frequency × season interactions were detected for butyrate (*p* = 0.032) and valerate (*p* = 0.008; Figure 4), and a pasture type × defoliation frequency × season interaction was detected for total VFAs (*p* = 0.016; Figure 5). NH_3_ concentration was affected by defoliation frequency (*p* = 0.003) and season (*p* < 0.001), with no significant interactions. Across pasture types, NH_3_ was higher at 150 GDD (28.4 mM) than at 300 GDD (26.3 mM), and spring values were higher than those in the other seasons.

The molar proportion of acetic acid was highest in the Msp pasture (67.01 mol 100 mol^−1^) compared to Lp (65.77 mol 100 mol^−1^), while Bv and LpBv had intermediate values. The molar proportion of propionic acid was higher in the Lp and Bv pastures (19.68 and 19.12 mol 100 mol^−1^, respectively) than in Msp (18.25 mol 100 mol^−1^). The Bv pasture had the highest proportions of isobutyric and isovaleric acids (1.05 and 1.79 mol 100 mol^−1^, respectively) compared to Lp, with LpBv and Msp at intermediate values. The 150-GDD defoliation frequency was associated with higher proportions of both branched-chain VFAs (isobutyric: 1.05 mol 100 mol^−1^; isovaleric: 1.81 mol 100 mol^−1^).

Both butyric and valeric acids followed a similar seasonal pattern within the DF × S interaction (Figure 4a,b), with higher proportions at 150 GDD; the difference between defoliation frequencies was significant in winter for butyric acid and in all seasons except spring for valeric acid.

The three-way interaction (Figure 5) indicated that total VFA concentrations were comparable among pastures in summer at both defoliation frequencies. In autumn, the Lp pasture produced higher total VFAs at 150 GDD than the other pastures, whereas at 300 GDD all pastures were similar. In winter, VFA concentrations were similar among pastures at 150 GDD, but Lp was higher than Bv at 300 GDD. In spring, total VFAs were highest overall, with Lp at 150 GDD significantly higher than at 300 GDD, while the other pastures remained stable across defoliation frequencies.

The acetate ratio was significantly affected by pasture type (*p* = 0.004) and season (*p* < 0.001), but not by DF (*p* = 0.246). None of the two-way or three-way interactions were significant (*p* > 0.05), indicating that the effects of species, defoliation frequency, and season were largely independent.

Least-squares means showed that Msp had the highest acetate:propionate ratio (3.71 ± 0.06), followed by Bv (3.49 ± 0.06), LpBv (3.48 ± 0.06), and Lp (3.37 ± 0.06), with Msp being significantly higher than Bv, Lp, and LpBv, whereas the remaining pastures did not differ significantly from each other. Across seasons, the highest acetate:propionate ratios were observed in summer (3.71 ± 0.06) and winter (3.69 ± 0.06), whereas lower values were observed in spring (3.36 ± 0.07) and autumn (3.29 ± 0.06).

### 3.4. In Vitro Gas and Methane Production

**Figure 6:**
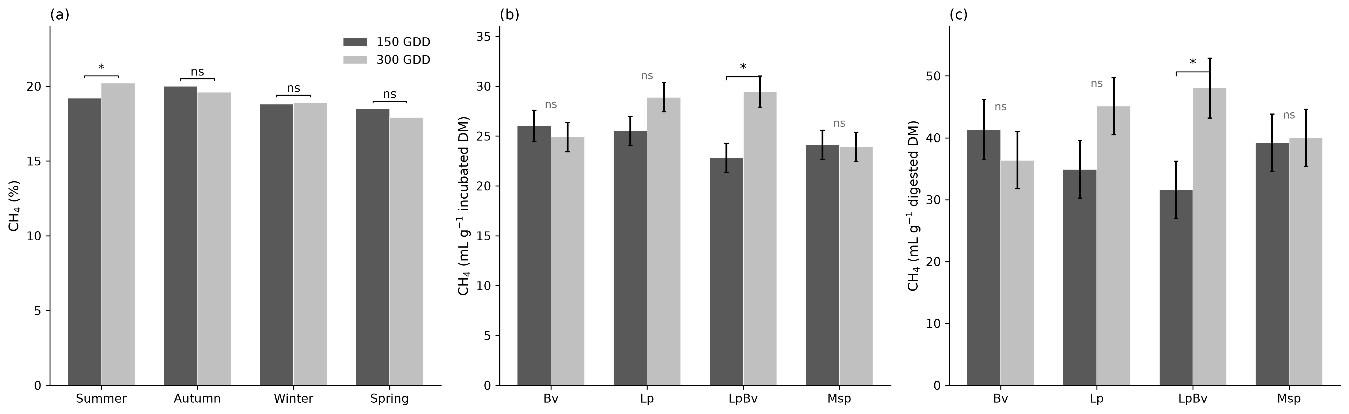
Interaction effects on in vitro methane production, (**a**) methane fraction in total gas at 48 h (DF × S), (**b**) mL of CH_4_ g^−1^ of incubated dry matter (P × DF) and (**c**) mL of CH_4_ g^−1^ of digested dry matter (P × DF). Bv: *Bromus valdivianus*; Lp: *Lolium perenne*; LpBv: Lp and Bv mixture; Msp: multispecies; GDD: growing degree days. In panel (**a**), an asterisk indicates a significant difference between 150 and 300 GDD within a season. In panels (**b**,**c**), an asterisk indicates a significant difference between 150 and 300 GDD within a pasture type (*p* < 0.05); ns indicates no significant difference. Error bars represent standard errors of the least-squares means.

Table 4 presents the effects of pasture type, defoliation frequency, season, and their interactions on total gas production and CH_4_ output. Season affected the CH_4_ proportion, CH_4_ per gram of incubated DM, CH_4_ per gram of incubated OM, and CH_4_ per gram of digested OM (*p* < 0.05). Defoliation frequency affected CH_4_ per gram of incubated DM (*p* = 0.043). Pasture type did not significantly affect any CH_4_ variable, although the CH_4_ proportion tended to differ among pasture types (*p* = 0.054), with Bv numerically higher than Lp. A DF × season interaction was detected for the CH_4_ proportion (*p* = 0.047; Figure 6a), and pasture type × DF interactions were detected for CH_4_ per gram of incubated DM and digested DM (*p* = 0.047; Figure 6b,c). Total gas production at 48 h was greater in Lp than in Bv and Msp and was greater at 300 than at 150 GDD (*p* < 0.001).

The DF × S interaction revealed that the effect of defoliation frequency on the CH_4_ proportion varied by season (Figure 6a); in summer, a higher percentage of CH_4_ was observed at 300 GDD compared to 150 GDD, whereas in spring, autumn, and winter, no differences between defoliation frequencies were detected.

The P × DF interaction for CH_4_ DMinc and CH_4_ DMdig (Figure 6b,c) reflected higher methane intensity at 300 GDD in the LpBv pasture, whereas Bv, Lp, and Msp showed comparable values across defoliation frequencies.

## 4. Discussion

### 4.1. Chemical Composition and In Vitro Ruminal Fermentation Kinetics

The chemical composition results indicate that defoliation frequency modulated the physiological maturity of the different species, whereas season influenced plant tissue structure [29]. Under more frequent defoliation (150 GDD), pastures remained at more juvenile physiological stages, with higher protein concentrations and lower structural-carbohydrate contents [30,31]. This pattern was evident in the monocultures and binary pasture. In contrast, Msp showed lower variability in chemical composition and fermentation response across defoliation intervals, which may reflect functional complementarity, phenological asynchrony, and differential growth rates among its constituent species [32,33]. This apparent stability refers to fermentation response rather than botanical composition, because Msp composition changed markedly across seasons and was dominated by chicory on most sampling dates (Figure 1). At the longer interval (300 GDD), greater tissue maturity increased structural-carbohydrate accumulation [30], as reflected by higher NDF and ADF. The magnitude of these effects varied seasonally, likely because temperature and growth conditions influence forage maturation and lignification [8,34,35].

In the first fermentation phase, associated with WSC fermentation, Lp showed the greatest gas production potential, consistent with its well-known capacity to accumulate fructans [36] and with the high WSC content recorded in this study. The higher k1 values observed for Msp and LpBv suggest that suggest that differences in the profile and availability of rapidly fermentable substrates, rather than WSC concentration alone, may have influenced the initial fermentation rate. Because individual carbohydrate fractions were not characterized, this interpretation should be considered tentative.

Total gas production over 48 h indicated greater fermentative potential for Lp and LpBv, consistent with the capacity of *L*. *perenne* to accumulate rapidly degradable WSCs, particularly fructans [37]. Conversely, the lower gas production observed in Msp, despite its moderate NDF concentration, indicates that total gas production did not depend solely on fiber content. The higher ash concentration commonly observed in herb-rich multispecies pastures [38] and the potential modulatory effects of plant secondary metabolites on rumen microbial activity [11] could have contributed. However, PSM concentrations and microbial populations were not measured, precluding direct attribution of the response to these mechanisms. In the subsequent fermentation phases, greater gas production was observed at 300 GDD, consistent with the greater accumulation of slowly degradable fiber associated with less frequent defoliation. Metabolically, increased fiber fermentation is associated with a higher relative proportion of acetate and, consequently, a potential increase in CH_4_ production [29]. In Msp, the lower contribution of the slowly degradable fraction and the stability across frequencies may be attributed to both its compositional diversity and the selective action of PSMs [11,37].

### 4.2. In Vitro Ruminal Metabolism

The main effects of pasture type and defoliation frequency on total VFA concentration were not significant; however, total VFAs varied through interactions with season, indicating that fermentation responses depended on the specific combination of pasture type, management, and season. Differences were also detected in individual VFA proportions. Lp had the lowest acetate-to-propionate ratio, consistent with a relatively greater propionate contribution and its role as a competitive H_2_ sink [6,34]. In contrast, Msp had a higher acetate-to-propionate ratio than Lp, but this did not translate into greater CH_4_ intensity. Potential PSM-mediated effects could contribute to this apparent decoupling, although this mechanism cannot be confirmed because PSMs and methanogenic populations were not measured.

Ruminal NH_3_ concentrations exceeded the minimum threshold required for adequate microbial protein synthesis and were within the expected range for temperate pastures in southern Chile [6,8]. Inclusion of broadleaf species did not reduce in vitro NH_3_ in the present study. The high crude-protein contribution of legumes and broadleaf species may have offset any potential protein-protective effects of PSMs. Chicory represented a greater proportion of Msp than plantain (Figure 1), whereas plantain is more consistently associated with reduced ruminal protein degradation [37]. In addition, red and white clover generally lack the condensed-tannin concentrations found in tanniferous legumes such as sainfoin and birdsfoot trefoil, which can reduce ruminal NH_3_ and improve nitrogen-use efficiency [39]. The higher NH_3_ concentration at 150 GDD was consistent with the greater protein and lower fiber concentrations of less mature forage. Excess ruminal NH_3_ may subsequently be converted to urea and excreted in urine, as outlined in the Introduction [5].

Regarding in vitro CH_4_ production, the significant pasture type × defoliation frequency and defoliation frequency × season interactions indicate that methane responses depended on both management and seasonal context. Pasture type did not significantly affect the CH_4_ proportion, although Bv tended to have a higher value than Lp, potentially reflecting its higher NDF and ADF and lower WSC concentrations. Previous studies have reported CH_4_ reductions when broadleaf species such as *Sanguisorba minor*, *Lotus pedunculatus*, or *Plantago lanceolata* were included in forage mixtures [17], as well as in some in vivo comparisons [40]. The absence of a comparable reduction in the present study may reflect differences in realized botanical composition, forage maturity, or concentrations of bioactive compounds, none of which can be isolated from the current data.

LpBv was the only pasture that showed a significant increase in CH_4_ intensity from 150 to 300 GDD. This response may reflect the combined contribution of the structural fiber supplied by *B*. *valdivianus* and the readily fermentable substrate supplied by *L*. *perenne* [41]. However, this interpretation remains a hypothesis because microbial pathways and substrate-specific fermentation were not measured. In Msp, CH_4_ intensity remained comparatively stable across defoliation intervals. This stability may be partly related to botanical diversity or potential modulatory effects of PSMs from broadleaf species, but direct attribution is not possible because PSM concentrations were not quantified. Although defoliation frequency did not consistently affect all CH_4_ metrics, advancing forage maturity can increase fiber-associated methanogenesis, whereas highly fermentable forage can also increase absolute CH_4_ formation by stimulating microbial activity [6,29,34].

Beyond environmental endpoints, defoliation frequency also altered forage nutritive value: the longer interval (300 GDD) lowered crude protein and raised NDF in the monocultures and binary pasture (Table 1), which could reduce milk production per hectare or increase reliance on concentrate supplementation [35]. Although a full economic analysis is beyond the scope of this in vitro study, any recommendation to extend the defoliation interval should therefore weigh the expected reduction in ruminal N losses against the cost of lower forage quality and potential additional concentrate use. This trade-off is consistent with the suggestion that environmental and productive objectives in pasture-based dairy systems need to be evaluated jointly [7,20].

Season further modified the methane response. In summer, the longer interval (300 GDD) increased the CH_4_ proportion in total gas, potentially reflecting greater structural-carbohydrate accumulation and a stronger acetogenic fermentation pattern [8,42]. In other seasons, the two intervals did not differ significantly. Seasonal changes in WSC availability and propionate formation may also alter hydrogen partitioning and methanogenesis [34].

The limited effects of Msp on NH_3_ and CH_4_ may be partly explained by its realized botanical composition. Chicory dominated the broadleaf fraction, whereas plantain—the species more consistently associated with reduced ruminal protein degradation—was present at low proportions across most seasons and defoliation intervals. Frequent or intensive defoliation can reduce plantain persistence by depleting root carbohydrate reserves [43,44]. Chicory contains several bioactive compounds, but their effects on ruminal proteolysis and methanogenesis are less consistently demonstrated and may vary seasonally [45]. Because PSM concentrations were not quantified, fermentation responses cannot be attributed directly to specific compounds. Additional methodological limitations are that pooling rumen fluid from the two donor cows prevented estimation of donor-specific variability, and pooling technical replicate vessels prevented estimation of vessel-level variability for VFAs, NH_3_, pH, and degradability. Future research should identify pasture species and combinations that persist under local management while producing reproducible fermentation effects [20].

## 5. Conclusions

Botanical composition alone did not guarantee lower CH_4_ production. Pasture type, defoliation frequency, and season jointly influenced in vitro fermentation, and no pasture treatment consistently reduced methane output. Although the main effects of pasture type and defoliation frequency on total VFA concentration were not significant, total VFAs varied through interactions with season; therefore, fermentation responses depended on the specific treatment and seasonal context.

Monocultures and the binary pasture were more responsive to defoliation management than Msp, which showed comparatively stable chemical and fermentative responses across intervals. Across pasture types, NH_3_ concentration was lower at 300 than at 150 GDD, indicating a potential reduction in ruminal nitrogen losses. However, the longer interval increased the CH_4_ proportion in summer and increased CH_4_ intensity in LpBv, while no treatment consistently lowered CH_4_. Extending the defoliation interval also lowered CP and increased NDF in the monocultures and binary pasture, which could affect animal performance and increase reliance on concentrate supplementation. Management recommendations should therefore balance potential nitrogen-related environmental benefits against forage-quality and feeding-cost trade-offs.

The limited fermentation response of Msp was likely related, at least in part, to its realized botanical composition: chicory dominated the broadleaf fraction, whereas plantain remained scarce. Multispecies pasture design should therefore prioritize species that persist and express relevant bioactive compounds under local conditions rather than relying on diversity alone. Because PSMs were not quantified, donor-cow inocula were pooled, and technical replicate vessels were pooled for several end points, specific mechanisms and some sources of biological and analytical variability could not be estimated. Future work should quantify PSMs, evaluate defoliation strategies that favor plantain persistence, and validate these findings in vivo together with an economic assessment of proposed management changes.

## Figures and Tables

**Table 1:** Effects of season and the pasture type × defoliation frequency interaction on pasture chemical composition (% DM, unless otherwise stated).

Pasture	DF	CP (%)	ME (Mcal kg DM^−1^)	NDF (%)	ADF (%)	SP (%)	WSC (g kg^−1^)	DV (%)	Ash (%)
Bv	150	23.0 ^ab^	2.5	56.5	27.4 ^b^	10.5 ^a^	120.5 ^c^	60.9	19.1
	300	17.5 ^d^	2.4	59.8	32.7 ^a^	7.2 ^de^	128.8 ^bc^	58.7	17.0
Lp	150	20.7 ^c^	2.6	54.3	26.0 ^bc^	9.1 ^bc^	146.9 ^ab^	65.1	17.2
	300	15.9 ^d^	2.5	54.8	27.5 ^b^	6.8 ^e^	170.3 ^a^	65.5	16.4
LpBv	150	21.5 ^bc^	2.5	54.5	26.6 ^b^	10.0 ^ab^	130.2 ^bc^	61.9	19.8
	300	16.8 ^d^	2.5	58.7	31.8 ^a^	7.0 ^e^	144.3 ^bc^	62.7	15.9
Msp	150	23.8 ^a^	2.5	44.6	22.4 ^d^	9.5 ^abc^	139.1 ^bc^	64.6	17.2
	300	21.8 ^abc^	2.5	47.7	24.4 ^cd^	8.3 ^cd^	126.2 ^bc^	62.7	17.8
Season									
Winter		22.7 ^a^	2.7 ^a^	59.4 ^a^	28.9 ^a^	10.2 ^a^	161.5 ^a^	69.2 ^a^	14.7 ^b^
Autumn		19.6 ^bc^	2.5 ^b^	53.1 ^b^	26.4 ^b^	8.8 ^b^	148.9 ^a^	66.6 ^a^	15.9 ^b^
Spring		19.8 ^b^	2.5 ^b^	52.6 ^b^	26.1 ^b^	8.3 ^b^	127.8 ^b^	63.4 ^b^	14.8 ^b^
Summer		18.4 ^c^	2.3 ^c^	50.5 ^c^	28.0 ^a^	6.8 ^c^	115.0 ^b^	51.9 ^c^	24.7 ^a^
		*p*-value							
P		<0001	<0.001	<0.001	<0.001	0.003	<0.001	<0.001	0.544
DF		<0.001	0.981	<0.001	<0.001	<0.001	0.035	0.32	0.023
S		<0.001	<0.001	<0.001	<0.001	<0.001	<0.001	<0.001	<0.001
P × DF		0.003	0.347	0.107	<0.001	<0.001	0.010	0.346	0.113
P × S		<0.001	<0.001	<0.001	0.005	<0.001	0.001	0.058	0.010
DF × S		<0.001	<0.001	0.166	<0.001	0.003	<0.001	<0.001	<0.001
P × DF × S		0.367	0.198	0.967	0.735	0.345	0.360	0.552	0.389

Within each factor and response variable, least-squares means with different superscript letters differ at *p* < 0.05; DF: defoliation frequency (growing degree days); CP: crude protein (%); ME: metabolizable energy (Mcal kg^−1^ DM); NDF: neutral detergent fiber (%); ADF: acid detergent fiber (%); SP: soluble protein (%); WSC: water-soluble carbohydrates (g kg^−1^ DM); DV: digestibility value (%); Ash: total ash (%); DM: dry matter; Bv: *B. valdivianus* monoculture; Lp: *L. perenne* monoculture; LpBv: binary pasture of *L. perenne* and *B. valdivianus*; Msp: multispecies pasture; P: pasture type; S: season; P × DF: pasture and defoliation frequency interaction; P × S: pasture and season interaction; DF × S: defoliation frequency and season interaction; P × DF × S: three-way interaction.

**Table 2:** Effect of pasture type, defoliation frequency, and season on in vitro fermentation kinetics.

	A1	k1	L1	A2	k2	L2	A3	k3	L3
Pasture									
Bv	22.42 ^b^	1.13 ^b^	0.45 ^b^	114.60	0.25 ^b^	4.70 ^ab^	67.74 ^a^	0.11	15.51
Lp	35.53 ^a^	1.36 ^ab^	0.53 ^ab^	115.33	0.27 ^ab^	4.10 ^ab^	70.12 ^a^	0.11	13.34
LpBv	27.2 ^ab^	1.46 ^a^	0.71 ^a^	112.56	0.27 ^ab^	4.85 ^a^	72.79 ^a^	0.12	14.59
Msp	31.98 ^ab^	1.46 ^a^	0.44 ^b^	104.40	0.30 ^a^	3.75 ^b^	49.90 ^b^	0.12	12.91
DF									
150	30.54	1.45	0.59	106.52	0.29	4.17	63.08	0.12	13.85
300	28.03	1.26	0.48	116.93	0.26	4.53	67.20	0.11	14.32
Season									
Winter	24.01 ^b^	1.49 ^ab^	0.90 ^a^	124.13 ^a^	0.28	5.68 ^a^	77.83 ^a^	0.13 ^a^	14.78
Autumn	25.67 ^b^	1.12 ^c^	0.57 ^b^	111.99 ^ab^	0.27	3.83^b^	82.85 ^a^	0.11 ^bc^	12.78
Spring	41.33 ^a^	1.17 ^bc^	0.43 ^bc^	108.35 ^ab^	0.26	3.70 ^b^	50.74 ^b^	0.10 ^c^	14.25
Summer	26.13 ^b^	1.64 ^a^	0.24 ^a^	102.42 ^b^	0.28	4.20 ^b^	49.12 ^b^	0.12 ^ab^	14.54
	*p*-value								
P	0.016	0.027	0.014	0.481	0.029	0.025	0.006	0.708	0.044
DF	0.401	0.034	0.070	0.062	0.070	0.211	0.395	0.401	0.502
S	<0.001	<0.001	<0.001	0.041	0.594	<0.001	<0.001	<0.001	0.124
P × DF	0.830	0.958	0.391	0.154	0.044	0.610	0.049	0.315	0.285
P × S	0.988	0.003	0.205	0.908	0.335	0.971	0.214	0.691	0.825
DF × S	0.455	0.151	0.275	0.811	0.291	0.774	0.078	0.715	0.301
P × DF × S	0.978	0.777	0.669	0.956	0.889	0.885	0.414	0.643	0.500

Within each factor and response variable, least-squares means with different superscript letters differ at *p* < 0.05; A1: gas production from soluble carbohydrate fermentation (mL g^−1^ DM); k1: fermentation rate of soluble carbohydrates (h^−1^); L1: lag time for soluble carbohydrates (h); A2: gas production from rapidly available fiber (mL g^−1^ DM); k2: fermentation rate of rapidly available fiber (h^−1^); L2: lag time for rapidly available fiber (h); A3: gas production from slowly available fiber (mL g^−1^ DM); k3: fermentation rate of slowly available fiber (h^−1^); L3: lag time for slowly available fiber (h); Bv: *B. valdivianus* monoculture; Lp: *L. perenne* monoculture; LpBv: binary pasture of *L. perenne* and *B. valdivianus*; Msp: multispecies pasture; P: pasture type; S: season; P × DF: pasture and defoliation frequency interaction; P × S: pasture and season interaction; DF × S: defoliation frequency and season interaction; P × DF × S: three-way interaction.

**Table 3:** Effect of pasture type, defoliation frequency, and season on the concentration of ammonia, volatile fatty acids, and their ratios in vitro.

	NH_3_	TVFA	C2	C3	C4	C5	i-C4	i-C5	a:p
	(mM)		(mol/100 mol)						
Pasture									
Bv	27.8	86.03	66.42 ^ab^	19.12 ^a^	10.3	1.32	1.05 ^a^	1.79 ^a^	3.49 ^b^
Lp	27.3	92.62	65.77 ^b^	19.68 ^a^	10.65	1.29	0.96 ^b^	1.64 ^b^	3.37 ^b^
LpBv	27.5	84.74	66.40 ^ab^	19.16 ^a^	10.42	1.3	1.00 ^ab^	1.73 ^ab^	3.48 ^b^
Msp	26.8	90.55	67.01 ^a^	18.25 ^b^	10.72	1.32	0.99 ^ab^	1.71 ^ab^	3.71 ^a^
DF									
150	28.4	88.42	66.26	18.88	10.66	1.35	1.05	1.81	3.54
300	26.3	88.55	66.55	19.23	10.38	1.26	0.95	1.63	3.49
Season									
Summer	26.0^b^	63.94 ^c^	67.65 ^a^	18.39 ^c^	10.18 ^b^	1.22 ^b^	0.95 ^b^	1.61 ^b^	3.71 ^a^
Autumn	26.5^b^	87.85 ^b^	63.65 ^c^	19.39 ^ab^	12.47 ^a^	1.46 ^a^	1.09 ^a^	1.95 ^a^	3.29 ^b^
Winter	26.5^b^	90.17 ^b^	68.54^a^	18.69 ^bc^	9.35 ^a^	1.14 ^c^	0.84 ^c^	1.44 ^c^	3.69 ^a^
Spring	30.4^a^	111.97 ^a^	65.77 ^b^	19.74 ^a^	10.09 ^b^	1.41 ^a^	1.12 ^a^	1.87 ^a^	3.36 ^b^
	*p*-value								
P	0.702	0.126	0.030	<0.001	0.111	0.389	0.016	0.019	0.004
DF	0.003	0.965	0.321	0.073	0.043	<0.001	<0.001	<0.001	0.246
S	<0.001	<0.001	<0.001	<0.001	<0.001	<0.001	<0.001	<0.001	<0.001
P × DF	0.940	0.078	0.876	0.813	0.945	0.640	0.724	0.665	0.827
P × S	0.802	0.044	0.434	0.679	0.460	0.121	0.303	0.263	0.601
DF × S	0.833	0.031	0.087	0.250	0.032	0.008	0.772	0.578	0.234
P × DF × S	0.999	0.016	0.272	0.536	0.735	0.304	0.576	0.558	0.457

Within each factor and response variable, least-squares means with different superscript letters differ at *p* < 0.05; TVFA: total volatile fatty acids; C2: acetic acid; C3: propionic acid; C4: butyric acid; C5: valeric acid; i-C4: isobutyric acid; i-C5: isovaleric acid; DF: defoliation frequency (growing degree days); Bv: *B. valdivianus* monoculture; Lp: *L. perenne* monoculture; LpBv: binary pasture of *L. perenne* and *B. valdivianus*; Msp: multispecies pasture; P: pasture type; S: season; P × DF: pasture and defoliation frequency interaction; P × S: pasture and season interaction; DF × S: defoliation frequency and season interaction; P × DF × S: three-way interaction.

**Table 4:** Effect of pasture type, defoliation frequency, and season on in vitro gas and methane production at 48 h.

	TGP	CH_4_ (%)	CH_4_ DM_inc_	CH_4_ DM_dig_	CH_4_ OM_inc_	CH_4_ OM_dig_
Pasture						
Bv	201.3 ^bc^	19.7	25.5	38.9	33.9	42.6
Lp	219.6 ^a^	18.9	27.2	40.0	35.3	42.8
LpBv	212.0 ^ab^	19.0	26.1	39.8	34.2	42.4
Msp	188.5 ^c^	19.1	24.0	39.6	31.6	40.4
DF						
150	196.7	19.1	24.6	36.7	32.7	40.4
300	214.0	19.1	26.8	42.4	34.8	43.7
Season						
Summer	175.2 ^b^	19.7 ^a^	23.7 ^b^	44.6	34.5 ^a^	47.6 ^a^
Autumn	226.6 ^a^	19.8 ^a^	28.7 ^a^	38.9	36.7 ^a^	42.4 ^ab^
Winter	227.1 ^a^	18.8 ^b^	28.5 ^a^	35.9	35.6 ^a^	40.0 ^ab^
Spring	192.6 ^b^	18.2 ^b^	21.9 ^b^	39.5	28.1 ^b^	38.2 ^b^
	*p*-value					
P	<0.001	0.054	0.186	0.993	0.227	0.849
DF	<0.001	0.998	0.043	0.050	0.111	0.127
S	<0.001	<0.001	<0.001	0.196	<0.001	0.020
P × DF	0.080	0.658	0.047	0.047	0.086	0.069
P × S	0.591	0.761	0.165	0.939	0.200	0.624
DF × S	0.156	0.047	0.193	0.322	0.285	0.431
P × DF × S	0.691	0.773	0.579	0.917	0.573	0.848

Within each factor and response variable, least-squares means with different superscript letters differ at *p* < 0.05; TGP: total gas production at 48 h (mL g^−1^ DM); CH_4_: proportion of CH_4_ in total gas; CH_4_ DM_inc_: mL of CH_4_ g^−1^ of incubated dry matter; CH_4_ DM_dig_: mL of CH_4_ g^−1^ of digested dry matter; CH_4_ OM_inc_: mL of CH_4_ g^−1^ of incubated organic matter; CH_4_ OM_dig_: mL of CH_4_ g^−1^ of digested organic matter; Bv: *B. valdivianus* monoculture; Lp: *L. perenne* monoculture; LpBv: binary pasture of *L. perenne* and *B. valdivianus*; Msp: multispecies pasture; P: pasture type; S: season; P × DF: pasture and defoliation frequency interaction; P × S: pasture and season interaction; DF × S: defoliation frequency and season interaction; P × DF × S: three-way interaction.

## Data Availability

The original data presented in the study are openly available in Zenodo at https://doi.org/10.5281/zenodo.21242059.
